# Essential role of conserved DUF177A protein in plastid 23S rRNA accumulation and plant embryogenesis

**DOI:** 10.1093/jxb/erw311

**Published:** 2016-08-29

**Authors:** Jiani Yang, Masaharu Suzuki, Donald R. McCarty

**Affiliations:** ^1^Plant Molecular and Cellular Biology Program, University of Florida, Gainesville, FL 32611, USA; ^2^Horticultural Sciences Department, University of Florida, Gainesville, FL 32611, USA

**Keywords:** *Arabidopsis thaliana*, background-dependent phenotype, chloroplast ribosome, DUF177, embryogenesis, *Zea mays.*

## Abstract

DUF177 proteins are nearly universally conserved in bacteria and plants except the Chlorophyceae algae. Thus far, *duf177* mutants in bacteria have not established a function. In contrast, *duf177a* mutants have embryo lethal phenotypes in maize and Arabidopsis. In maize inbred W22, *duf177a* mutant embryos arrest at an early transition stage, whereas the block is suppressed in the B73 inbred background, conditioning an albino seedling phenotype. Background-dependent embryo lethal phenotypes are characteristic of maize plastid gene expression mutants. Consistent with the plastid gene expression hypothesis, quantitative real-time PCR revealed a significant reduction of 23S rRNA in an *Escherichia coli duf177* knockout. Plastid 23S rRNA contents of *duf177a* mutant tissues were also markedly reduced compared with the wild-type, whereas plastid 16S, 5S, and 4.5S rRNA contents were less affected, indicating that DUF177 is specifically required for accumulation of prokaryote-type 23S rRNA. An AtDUF177A–green fluorescent protein (GFP) transgene controlled by the native *AtDUF177A* promoter fully complemented the Arabidopsis *atduf177a* mutant. Transient expression of AtDUF177A–GFP in *Nicotiana benthamiana* leaves showed that the protein was localized in chloroplasts. The essential role of DUF177A in chloroplast–ribosome formation is reminiscent of IOJAP, another highly conserved ribosome-associated protein, suggesting that key mechanisms controlling ribosome formation in plastids evolved from non-essential pathways for regulation of the prokaryotic ribosome.

## Highlight

A conserved role is established for DUF177A protein in prokaryote-type 23S rRNA accumulation, which is required for plant embryogenesis but non-essential for the survival of bacteria.

## Introduction

The plastid genome is derived from a cyanobacterial endosymbiont (Gould *et al.*, 2008). Hence, plastids have prokaryote-type ribosomes (70S) comprised of a small subunit (30S) that contains a 16S rRNA and a large subunit (50S) that contains 23S, 5S, and 4.5S rRNAs (Harris *et al.*, 1994). Plastid ribosomal proteins (PRPs) that are conserved in bacteria include 31 large subunit proteins and 21 proteins of the small subunit (Yamaguchi and Subramanian, 2000; Yamaguchi *et al.*, 2000). In the course of plant evolution, most *PRP* genes have transferred to the nuclear genome through a process of plastid genome reduction. The subset of genes retained in the plastid genome, which includes genes encoding 9 large and 12 small subunit proteins, is largely conserved among seed plants (Fleischmann *et al.*, 2011). An exception is transfer of the plastid *Rpl32* gene to the nucleus in *Populus* (Ueda *et al.*, 2007).

The consequences of disruption of plastid ribosome function also vary among species. In Arabidopsis, mutations in nuclear-encoded *PRP* genes typically have embryo lethal (emb) phenotypes. At least 14 PRPs (4 small subunit proteins and 10 ribosomal large subunit proteins) are required for normal embryogenesis (Bryant *et al.*, 2011; Romani *et al.*, 2012). Interestingly, mutant phenotypes of corresponding bacterial genes are a poor predictor of essentiality for embryogenesis in plants. For example, loss-of-function mutants of *RPS9*, *RPS20*, *RPL1*, *RPL21*, *RPL27*, *RPL31*, and *RPL35* in *Escherichia coli* are viable (Baba *et al.*, 2006; Shoji *et al.*, 2011), but mutations in the corresponding Arabidopsis *PRP* genes cause embryo lethality, indicating that these PRPs are essential in plants but not in bacteria (Bryant *et al.*, 2011; Romani *et al.*, 2012). Several plastid-encoded genes (*accD*, *ycf1*, and *ycf2*) have been shown to be essential for cell viability (Drescher *et al.*, 2000; Shikanai *et al.*, 2001; Cahoon *et al.*, 2003; Kode *et al.*, 2005), suggesting that a requirement for expression of these essential genes is the basis for the embryo lethality of plastid ribosome mutants (Bryant *et al.*, 2011).

Consistent with the essential gene hypothesis, in maize and other grasses that have lost *accD*, *ycf1*, and *ycf2* genes from the plastid genome (Konishi and Sasaki, 1994; Martin and Herrmann, 1998; Wicke *et al.*, 2011; Vries *et al.*, 2015), *PRP* genes and other genes implicated in plastid ribosome formation are typically non-essential for embryogenesis (Hess *et al.*, 1994; Zubko and Day, 1998; Asakura and Barkan, 2006). However, a re-assessment of the role of plastids in maize embryogenesis has been spurred by recent studies showing that the developmental fate of plastid ribosome mutants in maize is dependent on genetic background. In certain non-permissive genetic backgrounds (e.g. W22 inbred), mutations in the nuclear-encoded plastid ribosomal proteins, PRPL35 (Magnard *et al.*, 2004) and PRPS9 (*lem1*; Ma and Dooner, 2004); PPR8522 (*emb8522*; Sosso *et al.*, 2012); plastid translation initiation factor (*tif3*; Shen *et al.*, 2013); plastid ribosome assembly regulator, WHIRLY1 (*why1*; Zhang *et al.*, 2013); and EMB14 (*emb14*; Li *et al.*, 2015) uniformly block embryo development at an early transition stage. In contrast, in the permissive B73 inbred background, the emb phenotypes of *emb8522*, *tif3*, *why1*, and *emb14* mutants are suppressed (Sosso *et al.*, 2012; Zhang *et al.*, 2013) to condition an albino seedling phenotype. The conditional emb phenotype associated with diverse plastid mutants in maize suggests that the essential plastid gene hypothesis may not fully account for the role of plastids in plant embryogenesis (Bryant *et al.*, 2011).

Determining the biological and biochemical functions of proteins with a conserved domain of unknown function (DUF) is a major challenge in genome and molecular biology (Bateman *et al.*, 2010; Mudgal *et al.*, 2015). Although genes encoding DUF177 proteins are found in nearly all sequenced bacterial and land plant genomes (Goodacre and Gerloff, 2014), knockout mutations in bacteria have thus far failed to establish a biological function (Akerley *et al.*, 2002; Gerdes *et al.*, 2003; Kobayashi *et al.*, 2003; Kang *et al.*, 2004; Baba *et al.*, 2006; Commichau *et al.*, 2013).

Here we show that genomes of most land plants encode two distinct DUF177 domain proteins and that mutations in the *Duf177A* genes of maize and Arabidopsis have emb phenotypes indicating that DUF177A has a conserved, essential role in plants. In maize, the *duf177a* block in early embryogenesis is suppressible in a manner similar to the diverse class of mutants that have defects in plastid gene expression (Sosso *et al.*, 2012; Zhang *et al.*, 2013). In a non-permissive genetic background (W22), development of *duf177a* embryos is arrested at the early transition stage, whereas in the permissive background (B73) mutant seeds produce albino seedlings. Moreover, comparative analysis of bacterial genomes reveals a close association between *Duf177* and ribosomal protein *L32* genes, suggesting a functional relationship with the prokaryotic ribosome. Consistent with that hypothesis, analyses of *E. coli duf177* knockout and mutant maize tissues revealed marked reductions of prokaryote-type 23S rRNA accumulation. Transient expression of an AtDUF177A–green fluorescent protein (GFP) fusion protein in *Nicotiana benthamiana* leaf cells confirmed localization in chloroplasts with a punctate pattern, possibly in association with plastid nucleoids implicated in ribosome assembly. Our results indicate that DUF177 proteins specifically affect 23S rRNA accumulation in plastids as well as bacteria.

## Materials and methods

### Plant materials and growth conditions

The *duf177a-umu1* and *duf177a-umu2* alleles were isolated from the UniformMu (W22) transposon population (McCarty *et al.*, 2005). Maize plants were grown at the University of Florida Plant Science Research and Education Unit in Citra, FL during the spring and autumn seasons or sown in a winter greenhouse with supplemental light (16/8h light/dark cycle).

Seeds of the *atduf177a* T-DNA insertion line SALK_024559 obtained from the Arabidopsis Biological Resource Center (http://abrc.osu.edu/) were stratified at 4 °C in the dark for 2 d, sterilized, and plated on media containing 1× Murashige and Skoog salts, 0.05% MES, 1% sucrose, and 0.15% phytagel (Sigma). Seedlings were incubated in continuous light for 10 d at 22 °C, then transferred to soil and grown in a growth chamber under continuous light at ~22 °C for 4–6 weeks.

### Light microscopy of cytological sections

Developing wild-type and *duf177a* kernels were harvested at 7, 10, 14, and 20 days after pollination (DAP) from ears of self-pollinated heterozygous plants. Fixation, embedding, and sectioning were performed as described by Jackson (1991). Sections (8 µm) made with a Leitz 1512 microtome were stained with Johansen’s Safranin O and Fast Green and imaged with a Leica KL200 LED microscope.

### Genetic suppression of the emb phenotype

Heterozygous *duf177a/+* (W22 inbred) plants were reciprocally crossed with B73 inbred, and heterozygous F_1_ plants were self-pollinated to generate F_2_ populations. Seeds from segregating ears were classified by phenotype and counted for χ^2^ analysis. For seedling phenotypes, morphologically normal F_2_ seeds were germinated in soil in a greenhouse (16/8h light/dark cycle).

### RNA isolation and quantitative real-time PCR (qRT-PCR)

Total RNA was isolated using the Quick-RNA™ MiniPrep (Zymo Research) with In-column DNase I treatment according to the manufacturer’s instructions. First-strand cDNA was synthesized by SuperScript III reverse transcriptase (Invitrogen) using random hexamers for plastid rRNA measurements and oligo(dT) for mRNA analyses. For qRT-PCR, a SYBR^®^ Premix Ex Taq II (Tli RNaseH Plus) kit (TaKaRa) was used with the Applied Biosystems 7500 Fast Real-Time PCR System. In maize, the forward and reverse primer pair used for *Duf177A* gene expression was 5′-TCCTCAAGGTATATTTGCCAATTTCT/CAGTCGATATCTTGATCTCCATCCAT-3′, and for the plastid *Rpl32* gene it was 5′-AAAAACGTACTTCGATGTCAAAAA/AGAAAATGATCTTGATTTTGCTAAAGA-3′. For plastid 16S, 23S, 5S, and 4.5S rRNA levels, the forward and reverse primer pairs were 5′-TACCGTACTCCAGCTTGGTAGTTTC/GTAAGA CAGAGGATGCAAGCGTTAT-3′ (amplifying bases 881–1014), 5′-CCTATAACCATCTTTCGGCTAACCT/TAAGTCGATG GACAACAGGTCAATA-3′ (amplifying bases 1393–1485), 5′-AGAGGAACCACACCAATCCA/CCTACAGTATCGTCAC CGCA-3′ (amplifying bases 21–86), and 5′-CAAATCGTTCGTTCG TTAGG/GGTGTCAAGTGGAAGTGCAG-3′ (amplifying bases 4–64), respectively.

In *E. coli*, the forward and reverse primer pair used for the *rpmF* gene was 5′-GTACAACAGAATAAACCAACCCGTTC/AGGTGTTTT TCACCAGAAGTTTTGTC-3′. For *E. coli* 16S, 23S, and 5S rRNA levels, the forward and reverse primer pairs were 5′-TTAATACCTTTGCTCATTGACGTTAC/GGATTTCACATC TGACTTAACAAACC-3′, 5′-CTAAGGTCCCAAAGTCATGG TTAAGT/GACCAGTGAGCTATTACGCTTTCTTT-3′, and 5′-CGGTGGTCCCACCTGACC/GCCTGGCAGTTCCCTA CTCT-3′, respectively.

An absolute quantitative method was used for RNA analysis using an equal amount of total RNA per sample. Standard curves were generated from an independent cDNA sample subjected to the same RNA extraction and reverse transcription steps. An arbitrary copy number was assigned to the starting material of the dilution series, and the copy number of each point was calculated accordingly. To construct a standard curve, the log base 10 of the arbitrary copy number was taken for each dilution point and the Ct values of dilution points measured by qRT-PCR were fitted by linear regression. Then, the Ct values of samples were converted to the relative expression value based on the standard curve (Pfaffl, 2001).

### Vector construction and transformation

For transgenic complementation of Arabidopsis, Columbia-0 (Col-0) genomic fragments of 1964bp and 2973bp containing the *AtDuf177A* coding region as well as 910bp or 1919bp of upstream sequences, respectively, were PCR amplified by PrimeSTAR^®^ Max DNA polymerase (TaKaRa) using primer pairs 5′-CACCGTTGTTGTTGTTTGCTTCTTGT/GTTCCTTAGTCCCTCTTTTTGTTGC-3′ and 5′-CACCAAGAAGAAAGGGAACAAAATCA/GTTCCTTAGTCCCTCTTTTTGTTGC-3′, respectively. PCR products were cloned into the pGWB504 binary Gateway vector. *Agrobacterium tumefaciens* strain GV3101 was used to transform *atduf177a/+* plants by floral dip (Zhang *et al.*, 2006). Transformants were selected on 1× Murashige and Skoog plates containing hygromycin (25mg l^–1^) and carbenicillin (100mg l^–1^). Antibiotic-resistant seedlings were transferred to soil and grown in a growth chamber. T_1_ and T_2_ seedlings were genotyped by PCR using forward and reverse primers, 5′-GATAAACGCTTGATAAATTGCCTCTT/CAGTAACACCAACAAGATGAAGATG-3′ for the wild-type allele, 5′-GTTTGCTCTTTATCTTGTGTAGCTC/CAGTAACACCAACAAGATGAAGATG-3′ for the T-DNA insertion allele, and 5′-ATGTAACTGTGAAGTCTCGATACCC/AAGAAGATGGTGCGCTCCTGGACGTAG-3′ for detection of transgenes. Transgene expression was confirmed in independent lines by RT-PCR using primer pair, 5′-CACCATGTCTCTGGTTTGCTCTTTATC/CTTGTACAG CTCGTCCATGC-3′. To construct 35S-AtDUF177A–GFP for transient expression, a cDNA containing the AtDUF177A-coding region was amplified from leaf total RNA by PCR with primers, 5′-CACCATGTCTCTGGTTTGCTCTTTATC/GTTCCTTAGTCCCTCTTTTTGTTGC-3′, and cloned in a pGWB505 binary Gateway vector.

### Subcellular localization of AtDUF177A–GFP in tobacco leaf protoplasts

For subcellular localization of AtDUF177A–GFP, an overnight culture of *A. tumefaciens* GV3101 cells transformed with 35S-AtDUF177A–GFP was pelleted by centrifugation and suspended in inducing buffer (10mM MES, pH 5.6, 10mM MgCl_2_, 200 µM acetosyringone) to an OD_600_ of 0.1. Cells were injected into tobacco leaf epidermis with a syringe. Mesophyll protoplasts were isolated from infiltrated leaves 36–54h after injection as described in Yoo *et al.* (2007). Protoplasts were collected by centrifugation in a 15ml round bottom tube at 100 *g* for 2min, re-suspended in W5 solution [2mM MES (pH 5.7), 154mM NaCl, 125mM CaCl_2_, and 5mM KCl]. Protoplasts were imaged with an Olympus IX81 confocal fluorescence microscope.

## Results

### DUF177 proteins are universally conserved in bacteria and land plant genomes

**Fig. 1.**
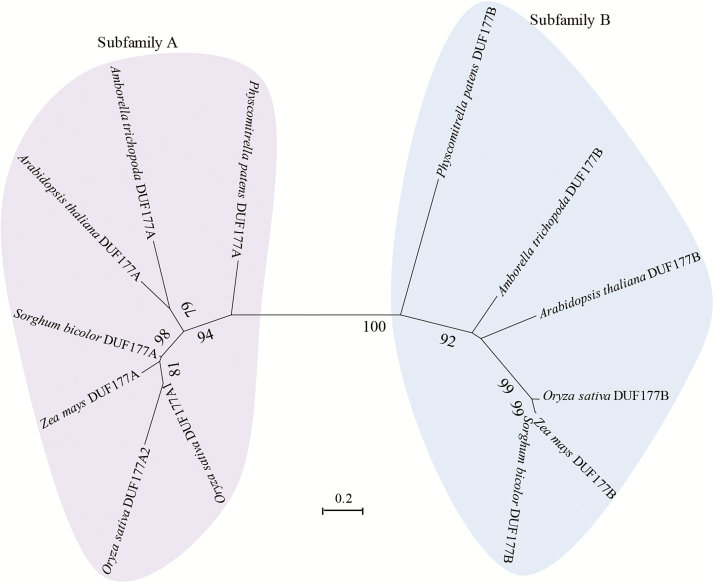
Unrooted tree of land plant DUF177 protein sequences. Protein sequences (Supplementary Data S1) were aligned by MUSCLE and used to construct a maximum likelihood tree based on the JTT matrix-based model by MEGA6 (Tamura *et al.*, 2013). Bootstrap support was based on 1000 iterations.

DUF177 proteins are found in nearly all sequenced bacteria and most plant genomes, but are not present in genomes of archaea, fungi, or animals (Goodacre and Gerloff, 2014). While DUF177 proteins were found to be broadly conserved in plants including diverse red algae and green algae genomes (Chlorophytes) as well as all sequenced land plant genomes (Streptophytae), exhaustive searches failed to detect DUF177 sequences in genomes of *Chlorella* and *Chlamydomonas* that represent the Chlorophyceae group of green algae. To gain understanding of the evolution of *Duf177* genes, a phylogenic analysis of DUF177 proteins from representative bacterial, algae, and plant genomes was performed (Fig. 1; Supplementary Data S1, S2; Supplementary Fig S1 at *JXB* online; Tamura *et al.*, 2013). The resulting tree revealed that all completely sequenced land plant genomes contain members of two distinct subfamilies, DUF177A and DUF177B (Fig. 1; Supplementary Data S1, S2; Supplementary Fig. S1), whereas genomes of red and green algae typically contain single DUF177 proteins that have varying affinities for the A and B groups and bacterial DUF177 proteins. While this relationship is consistent with the hypothesis that the DUF177A and DUF177B clades arose via duplication of an ancestral algal DUF177 gene, because some algal sequences grouped with bacterial DUF177 proteins, the alternative possibility that either DUF177A or DUF177B was acquired separately from bacteria by horizontal gene transfer could not be ruled out. In either case, the separation of DUF177A and DUF177B proteins most probably pre-dated the evolution of land plants. Although *Duf177A* and *Duf177B* genes are unlinked in most plant genomes, *Duf177A* (At3g19810) and *Duf177B* (At3g19800) genes are adjacent in the Arabidopsis genome.

More detailed sequence analysis of plant DUF177-containing proteins revealed conserved features of the DUF177 domain (Supplementary Fig. S2). The conserved amino acid residues in plant DUF177 domain sequences fell primarily into two clusters located in the N-terminal and middle regions of DUF177, respectively. A pair of cysteine motifs, C-X_(2)_-C and C-X_(3)_-C, located in cluster 1 (amino acids 13–16) and cluster 2 (amino acids 109–113), respectively, form a potential metal-binding structure. The DUF177A- and DUF177B-type proteins are distinguished by a 23 amino acid insertion (amino acids 53–76) in DUF177A.

### The *duf177a* mutant of maize has an emb phenotype

**Fig. 2.**
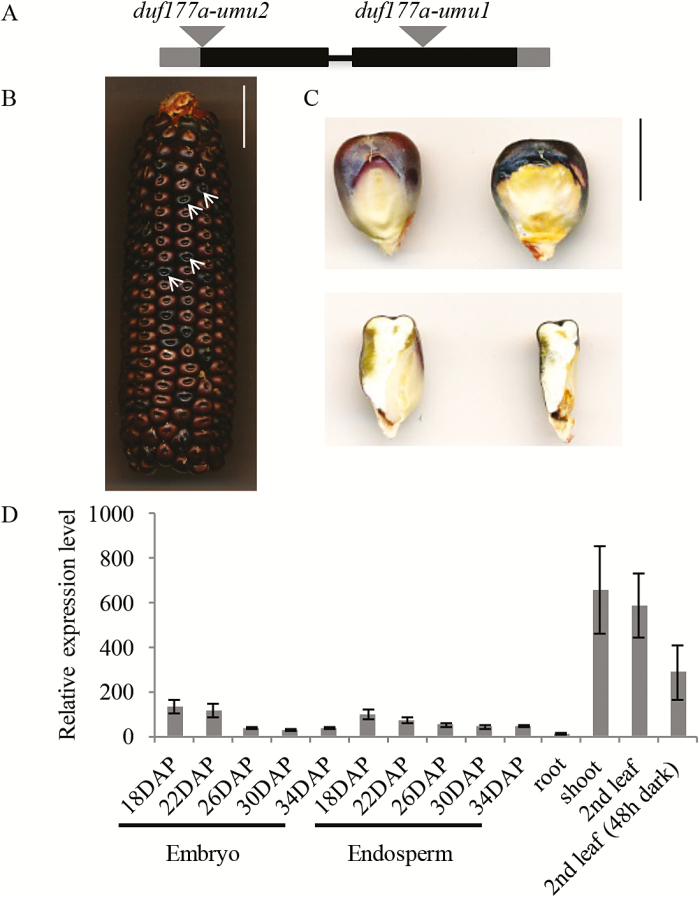
The embryo lethal phenotype of *duf177a* in a W22 inbred background. (A) Exon–intron structure of *Duf177A* consisting of two exons (solid rectangles) and one intron (horizontal line). The 5′- and 3′-untranslated regions are shown by gray rectangles. The gray triangles indicate the locations of Mu insertions. (B) A self-pollinated ear segregating *duf177a-umu1* mutant kernels (white arrows). Scale bar=2cm. (C) Seed phenotype of the *duf177a-umu1* mutant at maturity. Adgerminal views (top) and longitudinal sections (bottom) of wild-type (left) and mutant (right) kernels. Scale bar=1cm. (D) *Duf177A* gene expression profile determined by qRT-PCR. Relative expression of the *Duf177A* gene in endosperms and embryos sampled at various developmental stages and in roots, shoots, and second leaves from 2-week-old seedlings. For developing seed samples, error bars indicate the SEM of three technical replicates. For seedling samples, error bars indicate the SEM of three biological replicates.

The maize *Duf177A* gene (GRMZM2G433025) encodes a 293 amino acid protein containing a DUF177 between residues 152 and 289. Recessive mutations in *Duf177A* were identified in a screen of embryo-specific lethal seed mutants isolated from the UniformMu (W22 inbred) transposon population (McCarty *et al.*, 2005). Ears of self-pollinated heterozygotes segregated wild-type:emb seed in the expected Mendelian 3:1 ratio for a recessive mutant [588:187; *P* (χ^2^, 1 df)=0.58]. Mu-transposon insertions linked to the emb phenotype were identified using Mu-seq genotyping technology (McCarty *et al.*, 2013; Hunter *et al.*, 2014). The *duf177a-umu1* allele contains a Mu insertion in the second exon (+707) of GRMZM2G433025, while *duf177a-umu2* has a Mu insertion in the first exon (+46) (Fig. 2A). The allelic relationship between *duf177a-umu1* and *duf177a-umu2* was tested using reciprocal crosses of *duf177a-umu1/+* and *duf177a-umu2/+* plants. Segregation of the emb phenotype in ~25% of F_1_ seed confirmed that *duf177a* was responsible for the emb phenotype.

### *Duf177A* is broadly expressed during plant development

To characterize expression of *Duf177A* during normal development, mRNA was quantified by qRT-PCR in diverse maize tissues including hand-dissected embryos and endosperms harvested at different developmental stages, as well as root, young leaf, and shoot (emerging leaves covered by sheath) tissues sampled from 2-week-old seedlings. *Duf177A* expression was detected in all tissues tested though transcript levels varied (Fig. 2D). Expression of *Duf177A* was markedly higher in photosynthetic shoot and leaf tissues in comparison with root, embryo, and endosperm. The lack of a pronounced expression difference in embryo and endosperm indicated that the embryo-specific phenotype of the *duf177a* mutant was not due to embryo-specific expression of *Duf177A*. The qRT-PCR results were consistent with transcriptome data obtained from qTeller (http://qteller.com/) showing universal expression of *Duf177A* and high expression in green tissues.

### *duf177a* blocks development at an early transition stage of embryogenesis

At maturity, *duf177a* mutant seed contains only a small remnant of dark brown necrotic tissue at the position of the embryo, whereas the mutant endosperm forms a cavity on the germinal face in the space where the embryo would normally develop (Fig. 2C). Except for the presence of an empty embryo cleft, the endosperm of the *duf177a* kernel was fully developed and morphologically normal, with slightly more intense anthocyanin pigmentation than the wild-type (Fig. 2B, C), while the endosperm dry weight of *duf177a* mutant kernels was 14–25% lower compared with the wild-type (Supplementary Fig. S3). In these respects, *duf177a* has a typical maize emb phenotype as described in previous studies (Clark and Sheridan, 1991; Ma and Dooner, 2004; Magnard *et al.*, 2004; Sosso *et al.*, 2012; Shen *et al.*, 2013; Zhang *et al.*, 2013; Li *et al.*, 2015).

**Fig. 3.**
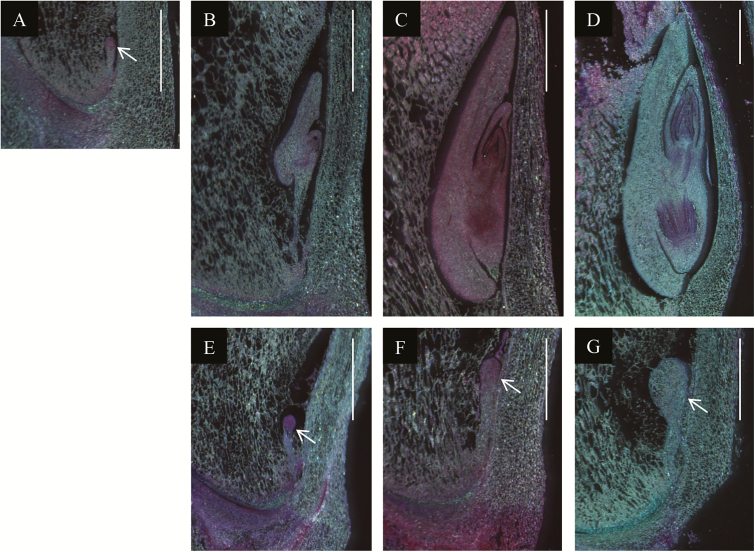
Embryo phenotype of the *duf177a* mutant. Wild-type (A–D) and *duf177a-umu1* mutant (E–G) embryos at 7 (A), 10 (B, E), 14 (C, F), and 20 (D, G) DAP were longitudinally sectioned for light microscopy. Embryos at the transition stage are indicated by white arrows. Scale bars=1mm.

Longitudinal sections of mature mutant kernels indicated that embryo development was uniformly arrested during early embryogenesis (Fig. 2C). To resolve the developmental stage at which embryogenesis is blocked, developing kernels from segregating ears of self-pollinated heterozygous plants were harvested at 7, 10, 14, and 20 DAP, embedded in paraffin, and sectioned for examination by light microscopy. In wild-type kernels, a proembryo with radially symmetric embryo proper and basal suspensor, typical of an early transition stage, was visible at 7 DAP (Fig. 3A). At 10 DAP, scutellum, coleoptile, and shoot and root meristem structures were well developed in wild-type embryos (Fig. 3B). By 14 DAP, the shoot apical meristem (SAM) had formed 2–3 primary leaves (Fig. 3C). At 20 DAP, 5–6 primary leaves formed in the embryo covering the SAM, and hypocotyl, radicle, and coleorhiza were well developed at this stage (Fig. 3D). The *duf177a* mutant embryos were indistinguishable from the wild-type at 7 DAP, whereas mutant embryos were clearly identifiable at 10 DAP, indicating that development was arrested at some point between 7 and 10 DAP (Fig. 3A, B, E). At 10 DAP, mutant embryos resembled normal, albeit slightly enlarged, early transition stage embryos with no evidence of apical meristem formation. By 14 DAP, the morphology of mutant embryos was basically unchanged except for increased size relative to 10 DAP embryos (Fig. 3F). At 20 DAP, the embryo proper of transition stage-like mutant embryos was further enlarged without an obvious increase in the size of the suspensor (Fig. 3G). These results indicated that *duf177a* mutant embryos do not progress beyond the early transition stage of embryogenesis. In this respect, the *duf177a* phenotype is similar to an emerging class of maize emb mutants implicated in disruption of plastid gene expression (Ma and Dooner, 2004; Magnard *et al.*, 2004; Sosso *et al.*, 2012; Shen *et al.*, 2013; Zhang *et al.*, 2013; Li *et al.*, 2015). In maize, the emb phenotypes of plastid gene expression mutants are suppressed in certain inbred backgrounds to condition an albino seedling phenotype (Sosso *et al.*, 2012; Zhang *et al.*, 2013).

### Genetic suppression of the *duf177a* emb phenotype

**Fig. 4.**
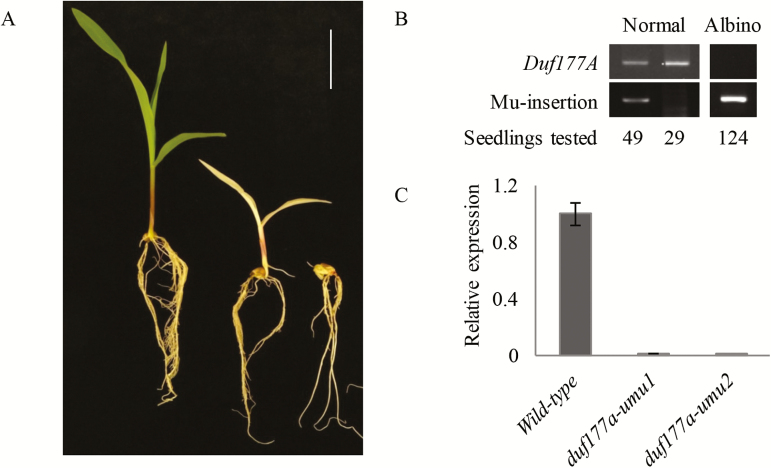
Segregation of *duf177a* seedling phenotypes in hybrid F_2_ progeny. (A) Seedling phenotypes of *duf177a-umu1* observed in the B73/W22 hybrid F_2_ progeny included normal green seedlings (left), morphologically normal albino seedlings (middle), and seedlings with abnormal shoot development (right). Scale bar=5cm. (B) PCR genotyping of albino F_2_ seedlings for *duf177a-umu1*. Duf177A F1/R1 primers amplify the wild-type *Duf177A* allele (upper panel) and Duf177A F1/TIR6 primers amplify the Mu insertion allele (lower panel). Numbers underneath indicate the number of individuals identified with each genotype. (C) qRT-PCR showing absence of *Duf177A* gene expression in albino seedling leaves. Total RNA samples extracted from wild-type and albino leaves from 2-week-old seedlings were analyzed. Error bars indicate the SEM of three biological replicates.

To test whether the *duf177a* emb phenotype was suppressible, heterozygous *duf177a/+* plants were crossed to the B73 inbred, a permissive genetic background for embryogenesis of mutants with defects in plastid gene expression (Sosso *et al.*, 2012; Zhang *et al.*, 2013). Among F_2_ progeny of the B73/W22 hybrid, segregation of normal to emb seed was significantly skewed from the Mendelian 3:1 ratio obtained in the W22 background (noted above) with an excess of normal seeds (*P*=9.26×10^−42^), consistent with suppression of embryo lethality (Table 1). The morphologically normal F_2_ seeds were then germinated to evaluate seedling phenotypes. Albino seedlings were produced by 11.52% of the morphologically normal seeds and 10.10% of total seeds (Fig. 4A; Table 1), whereas no albino seedling was detected among progeny of self-pollinated *duf177a/+* (W22) control plants. PCR genotyping showed that all of the albino F_2_ seedlings were homozygous for *duf177a* (Fig. 4B), confirming that the albino seedlings resulted from suppression of the *duf177a* emb phenotype. In addition, a few F_2_ seeds germinated to produce seedlings with abnormal shoot development (Fig. 4A). Genotyping confirmed that the abnormal seedlings were also homozygous *duf177a.* The emb, albino, and shootless seedling classes combined accounted for ~25% of the F_2_ progeny that were expected to be homozygous mutant.

**Table 1.** Segregation of emb and albino seedling phenotypes of F_2_ progeny in hybrid background

F1 parental genotype (*♀×*♂)	Total seed	Seed phenotype (%)		*P*-value of *X* ^*2*^ test (Expected ratio 3:1)	Seedling phenotype (%)		Total of emb and albino (%)
		Normal	emb		Normal	Albino	
B73*×duf177a/+*	1226	87.68	10.96	9.26×10^−42^ **	78.30	10.10	21.06
*duf177a/+×*B73	426	84.98	15.02	1.98×10^−42^ **	73.94	10.56	25.58

**P<0.01.

To test whether maternal effects contribute to the genetic background suppression of the emb phenotype of the *duf177a* mutant, crosses between heterozygous *duf177a/+* as female and B73 inbred as male were made. The emb phenotype of F_2_ populations was also suppressed to the albino seedling phenotype with a 10.56% segregation ratio of albino seedlings at the total seed base (Table.1). The results indicated that maternal effects had little or no influence on suppression of the emb phenotype.

Consistent with the genotyping results, qRT-PCR analyses failed to detect *Duf177A* transcripts in leaves of albino seedlings (Fig. 4C), indicating that the *duf177a-umu1* and *duf177a-umu2* alleles are null mutations.

### DUF177 is functionally associated with prokaryotic ribosomes

**Fig. 5.**
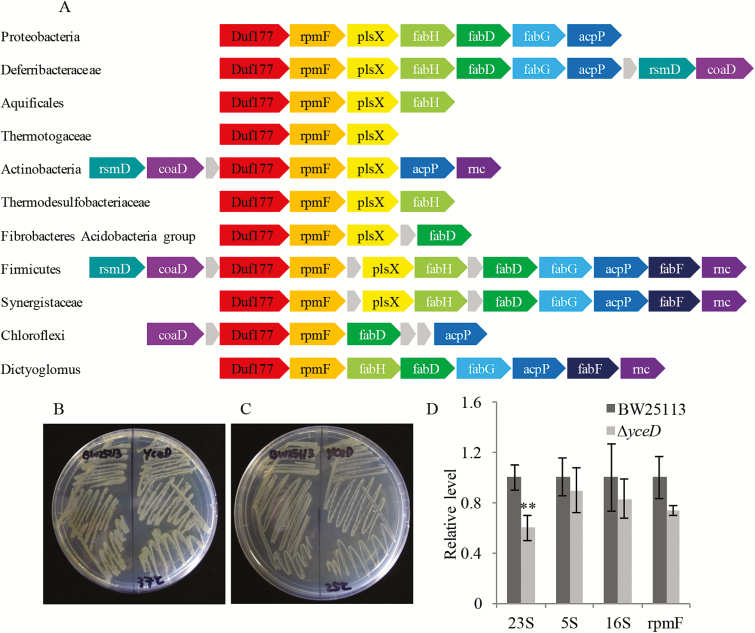
Association between bacterial DUF177 and prokaryotic ribosomes. (A) Bacterial *DUF177* genes function in a conserved operon. An association with ribosomal protein L32 (rpmF) and fatty acid biosynthetic genes is conserved in diverse groups of bacteria. plsX, fatty acid phospholipid synthesis protein; fabH, 3-oxoacyl-[acyl-carrier-protein] synthase III; fabD, malonyl CoA-acyl carrier protein transacylase; fabG, 3-oxoacyl-[acyl-carrier-protein] reductase; acpP, acyl carrier protein; fabF, 3-oxoacyl-[acyl-carrier-protein] synthase II; rsmD, ribosomal RNA small subunit methyltransferase D; rnc, ribonuclease III. Gray arrows represent either genes less conserved in a gene cluster or genes encoding uncharacterized proteins. Data are from the STRING database (Szklarczyk *et al.*, 2015). Growth of the *ΔyceD* mutant is comparable with that of the BW25113 (wild-type) at 37 °C (B) and 25 °C (C). (D) Relative accumulation of bacterial ribosome 23S, 5S, and 16S rRNAs and the relative expression of the *rpmF* gene in the wild-type (BW25113) and *ΔyceD* mutant. **P<0.01. Error bars indicate the SEM of three biological replicates.

A comparative genomic analysis utilizing the SEED (Overbeek *et al.*, 2005) and STRING (Szklarczyk *et al.*, 2015) databases was used to explore possible functions of DUF177 proteins. In the *E. coli* genome, the *Duf177* homolog *yceD* is located directly upstream of and co-transcribed with the *rpmF* gene that encodes ribosomal large subunit protein L32. This association is remarkably conserved in diverse bacterial clades including Proteobacteria, Deferribacteraceae, Aquificales, Thermotogaceae, Actinobacteria, Thermodesulfobacteriaceae, Fibrobacteres Acidobacteria group, Firmicutes, Synergistaceae, Chloroflexi, and Dictyoglomus (Fig. 5A). In *Clostridium thermosaccharolyticum*, DUF177 is fused with ribosomal protein L32, reinforcing a close functional relationship. An association of bacteria *DUF177* homologs with RNase III genes involved in rRNA processing is also detected in Actinobacteria, Firmicutes, Synergistaceae, and Dictyoglomus. In addition to being linked to genes involved in bacterial ribosome formation, *Duf177* clusters with genes involved in phospholipid synthesis (*plsX*; Lu *et al.*, 2006) and fatty acid elongation (*fabH*, *fabG*, *acpP*, and *fabF*; Rock and Jackowski, 2002).

However, in spite of being among the most broadly conserved bacterial genes known, there is no direct evidence that *Duf177* genes are essential in bacteria (Akerley *et al.*, 2002; Gerdes *et al.*, 2003; Kobayashi *et al.*, 2003; Kang *et al.*, 2004; Baba *et al.*, 2006; Commichau *et al.*, 2013). High-throughput analyses of *E. coli* gene knockouts grown on diverse media did not establish a well-defined phenotype for the *Duf177* homolog *yceD* (Baba *et al.*, 2006; Nichols *et al.*, 2011). Consistent with that study, we found that on rich media, growth of the *E. coli Duf177* knockout strain *ΔyceD* was similar to that of BW25113 (wild-type) at 37 °C and 25 °C (Fig. 5B, C). However, qRT-PCR quantification of the *rpmF* transcript and 5S, 23S, and 16S rRNAs in *ΔyceD* cultures showed that 23S rRNA content was decreased in *ΔyceD* compared with the wild-type, whereas the effects on 5S and 16S rRNAs and *rpmF* (L32) RNA levels were not statistically significant (Fig. 5D). Our qRT-PCR results are consistent with a specific role for DUF177 in synthesis, processing, and/or stability of 23S rRNA in bacteria.

### DUF177A is required for accumulation of 23S plastid rRNA

**Fig. 6.**
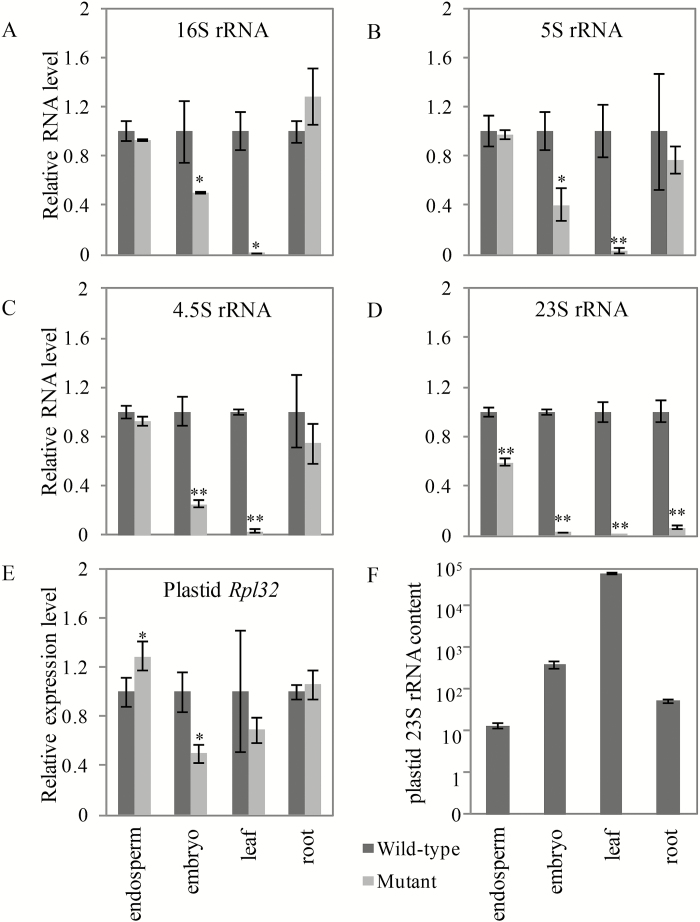
Plastid rRNA contents and plastid *Rpl32* gene transcript levels in *duf177a* tissues. Relative accumulation of plastid 16S (A), 5S (B), 4.5S (C), and 23S (D) rRNAs and relative expression of plastid *Rpl32* (E) in wild-type and *duf177a-umu1* endosperms (21 DAP), embryos (23 DAP), and leaves and roots of 2-week-old seedlings. (F) Relative plastid 23S rRNA contents of 21 DAP endosperms, 23 DAP embryos, and leaves and roots from 2-week-old seedlings.*P<0.05, **P<0.01. Error bars indicate the SEM of three biological replicates. (This figure is available in colour at *JXB* online.)

To address a possible role for maize DUF177A in plastid ribosome formation, rRNA components of plastid 70S ribosomes were quantified in mutant and wild-type tissues by qRT-PCR, a general method to detect the plastid rRNAs (Pyo *et al.*, 2013; Li *et al.*, 2015; Zhao *et al.*, 2016). Total RNA was extracted from both mutant and wild-type tissues, including 21 DAP endosperms of W22 inbred, and 23 DAP embryo and leaf and root tissues of 2-week-old F_2_ seedlings of the B73/W22 hybrid. The latter included viable embryos and albino seedlings that were homozygous *duf177a*. The qRT-PCR results showed that 23S rRNA contents of endosperm, embryo, leaf, and root tissues were significantly decreased in the mutant compared with the wild-type (Fig. 6D). In contrast, levels of 16S, 4.5S, and 5S rRNAs were less affected, showing significant reduction only in mutant embryos and albino leaves of B73/W22 F_2_ seedlings (Fig. 6A–C). The significant decrease of plastid 23S rRNA in all tissues tested indicated that DUF177A is specifically required for normal accumulation of 23S rRNA. Overall, the fold reduction of 23S rRNA content in the mutant was in proportion to the total amount of plastid 23S rRNA found in normal tissues (Fig. 6F). qRT-PCR analysis showed that the level of plastid 23S rRNA in leaves was ~ 150 times the amount found in embryos, and 1000-fold and 4000-fold greater than in roots and endosperm, respectively.

To examine further a potential functional association between DUF177A and RPL32 in plastids, transcript abundance of plastid *Rpl32* was analyzed in mutant and wild-type endosperm, embryo, and leaf and root tissues by qRT-PCR. Compared with the wild-type, the level of plastid *Rpl32* transcript was reduced by >2-fold in mutant embryos, whereas relative expression in mutant endosperms was slightly greater than in the wild-type. In contrast, *Rpl32* expression in roots and albino seedling leaves was not significantly affected by the *duf177a* mutant (Fig. 6E).

### DUF177A is required for Arabidopsis embryogenesis

**Fig. 7.**
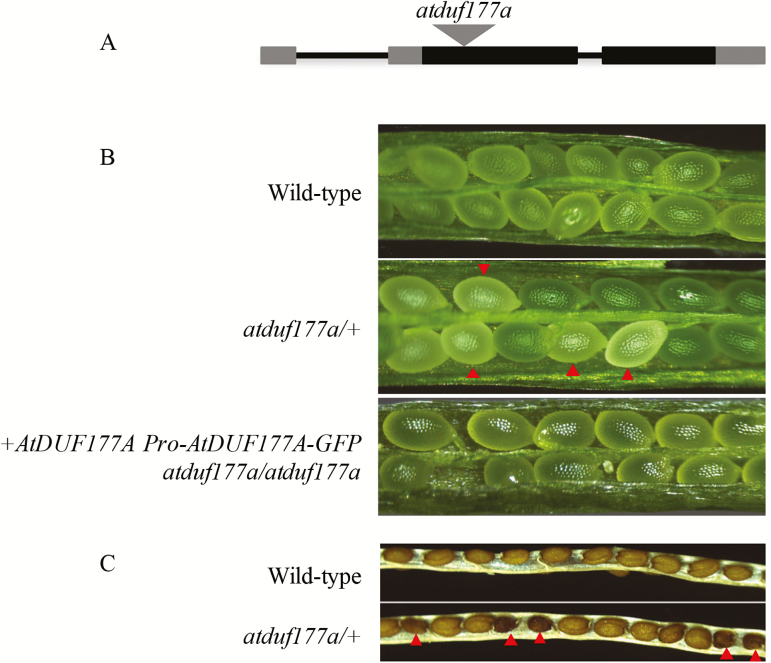
*atduf177a* mutant. (A) Exon structure of *AtDuf177A* (rectangles) and T-DNA insertion site (gray triangle). The 5′- and 3′-untranslated regions are shown by gray rectangles. (B) Green siliques of wild-type (upper panel) and a self-pollinated *atduf177a/+* plant (middle panel) segregating white seeds (red triangles) and a homozygous *atduf177a* plant (lower panel) carrying the plus 910bp AtDUF177A–GFP transgene. (C) Mature silique of a self-pollinated *atduf177a/+* plant segregating brown shriveled seeds (red triangles).

In order to determine whether the function of DUF177A is conserved in plants, a T-DNA insertion line (SALK_024559) that has an insertion located in the second exon of the Arabidopsis *Duf177A* (At3g19810) gene was characterized (Fig. 7A). Segregation of the insertion allele was analyzed by PCR genotyping progeny of self-pollinated *atduf177a/+* plants. A set of 91 progeny included 60 heterozygous individuals and 31 homozygous wild-type seedlings. The failure to detect plants homozygous for the T-DNA insertion suggested that the *atduf177a* mutant had a seed lethal phenotype. Consistent with that hypothesis, segregation of white emb seeds was observed in immature siliques of self-pollinated heterozygotes (Fig. 7B). At maturity, the defective seeds appeared dark brown and shrunken (Fig. 7C). Transmission bias was tested by making reciprocal crosses of heterozygous *atduf177a*/+ and wild-type Col-0 plants. PCR genotyping results showed that in both directions, 19 out of 40 progeny of (*atduf177a/+*)/Col-0 and 7 out of 13 Col-0/(*atduf177a/+*) carry the T-DNA insertion, which fit the expected 1 heterozygote:1 wild-type ratio for unbiased transmission through male and female gametophytes with *P*=0.75 and 0.78, respectively.

To confirm that the T-DNA insertion in *AtDuf177A* is the cause of the emb phenotype, transgenic experiments were performed to determine whether a wild-type *AtDuf177A* sequence was able to complement the emb phenotype. Col-0 genomic DNA fragments of 1964bp and 2973bp containing the *AtDUF177A* coding region plus 910bp and 1919bp of upstream sequence, respectively, were cloned into the pGWB504 vector to create a C-terminal GFP fusion protein. The constructs were introduced into plants heterozygous for the *atduf177a* mutant by floral dip (Zhang *et al.*, 2006), and transformed seedlings were selected based on resistance to hygromycin. Presence of the transgene was confirmed by PCR genotyping and by RT-PCR. Transformed seedlings were allowed to self-pollinate and the progeny were genotyped for the presence of the *atduf177a* T-DNA insertion. If complementation occurred, we expected to recover homozygous *atduf177a* mutant seedlings among the progeny. Complementation was confirmed for both constructs with at least two independent transgenic lines. A homozygous *atduf177a* mutant carrying the plus 910bp transgene is shown in Fig. 7B. Overall, 18.57% (44:237) of plants were homozygous mutant, consistent with the expected frequency of 20% (*P*=0.069). The expected frequency (3/15=20% of seedlings) is based on the assumption that if complementation occurs, 1/16 of seeds will not germinate to form seedlings. Seed and seedling phenotypes of the complemented homozygous *atduf177a* mutant were indistinguishable from those of the wild-type. These results confirmed that the *atduf177a* gene is responsible for the emb phenotype.

### Subcellular localization of AtDUF177A protein

**Fig. 8.**
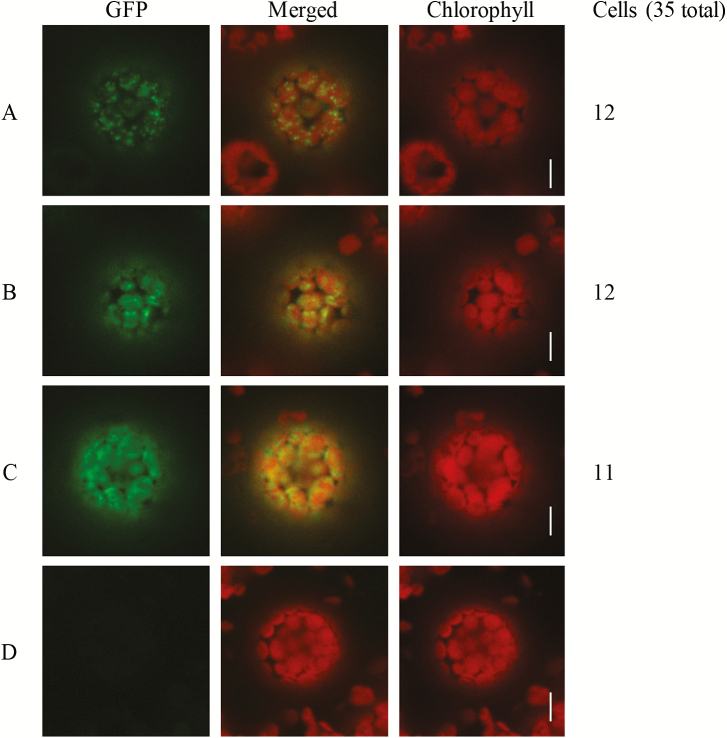
Subcellular localization of AtDUF177A. Confocal fluorescence detection of GFP in mesophyll protoplasts isolated from *N. benthamiana* leaf cells transformed with 35S-AtDUF177A–GFP (A–C) including punctate (A), intermediate (B), and filamentous (C) patterns of fluorescence, plus a non-transformed control (D). Numbers (right) indicate protoplasts observed in each class. Scale bars=10 μm.

Both maize and Arabidopsis DUF177A proteins are predicted to be targeted to either the chloroplast or mitochondria according to TargetP (Emanuelsson *et al.*, 2007). DUF177A was previously detected in the stroma proteomes of maize bundle sheath and mesophyll chloroplasts (Huang *et al.*, 2013). Although AtDUF177A–GFP expressed under the control of the native promoter was able to complement the *atduf177a* phenotype fully, we were unable consistently to detect or localize GFP fluorescence in transgenic seedlings. Interestingly, AtDUF177A has thus far been detected in chloroplast stroma proteomes of *clpr4-1* and *clpc1* mutants that are deficient in plastid ClpPR protease (Kim *et al.*, 2009) though not in the wild-type, suggesting that AtDUF177A accumulation may be limited by protein turnover. As an alternative approach, subcellular localization of the AtDUF177A–GFP fusion protein was analyzed by transient overexpression in *N. benthaminana* leaves via *Agrobacterium* infiltration. To enhance image resolution, mesophyll protoplasts were isolated from *N. benthaminana* leaves after infiltration. In agreement with the predicted localization, confocal microscope imaging showed that GFP fluorescence from AtDUF177A–GFP co-localized with chlorophyll autofluorescence in the *N. benthaminana* mesophyll protoplasts (Fig. 8). There is some evidence that ribosome assembly is associated with plastid nucleoids (Majeran *et al.*, 2012; Bohne, 2014). A set of 35 protoplasts that showed co-localization of GFP signal with the chlorophyll autofluorescence included 12 protoplasts in which GFP fluorescence had a punctate distribution within the chloroplast, a pattern that resembles the positioning of nucleoids before and after chloroplast division (Powikrowska *et al.*, 2014). About one-third (11 of 35) of the protoplasts showed a filamentous distribution of GFP signal at the chloroplast periphery, a pattern consistent with the distributions of nucleoids during early stages of chloroplast division (Terasawa and Sato, 2005). The remaining 12 protoplasts exhibited an intermediate pattern. However, analyses of maize (Majeran *et al.*, 2012) nucleoid proteomes did not detect enrichment of DUF177A in the nucleoid fraction.

## Discussion

Our results reveal an essential role for the highly conserved *Duf177A* gene in embryogenesis and chloroplast development. While DUF177 proteins are universally conserved in bacteria, high-throughput phenotyping studies (Baba *et al.*, 2006; Nichols *et al.*, 2011) and our targeted analyses of the *ΔyceD* mutant do not indicate an essential role for DUF177 in *E. coli*. In contrast, the emb phenotypes of *duf177a* mutants in maize and Arabidopsis demonstrate that *Duf177A* genes are required for embryogenesis in plants. Analysis of the albino seedling phenotype conditioned by genetic suppressors in maize further implicates DUF177A in accumulation of plastid 23S rRNA of the ribosome large subunit in non-photosynthetic plastids as well as chloroplasts. A conserved role in prokaryote-type ribosome large subunit formation is suggested by reduced accumulation of 23S rRNA in the *E. coli ΔyceD* mutant and in plastids of the maize *duf177a* mutant.

### DUF177A plays a role in plastid 23S rRNA accumulation

Our results implicate DUF177A in accumulation of plastid 23S rRNA in plants. Comparative genome analyses in bacteria reveal that *Duf177* genes strongly associated with the ribosomal protein L32 (*rpmF*) gene in a cluster that includes *plsX* involved in phospholipid synthesis (Lu *et al.*, 2006) and *fab* genes (*fabH*, *fabD*, and *fabG*) encoding fatty acid biosynthetic enzymes (Fig. 5A; Rock and Jackowski, 2002). In *E. coli*, *yceD* and *rpmF* comprise an operon transcribed from a promoter located upstream of the *yceD* gene (Tanaka *et al.*, 1989). The inclusion of *yecD* and *rpmF* within a polycistronic transcription unit implies co-ordinate regulation of *yceD* with ribosome assembly and protein translation. On the other hand, *rpmF*, *plsX*, and *fab* genes form a separate operon which co-ordinates protein translation with biosynthesis of cell membranes (Podkovyrov and Larson, 1995). Thus far, *yceD* has not been detected in the same operon with *plsX* and *fab* genes, implying that they are not strictly co-regulated. Our results reveal a significant decrease in 23S rRNA accumulation in the *ΔyceD* mutant, suggesting a specific function related to processing and/or stability of the 23S rRNA (Fig. 5D). Because *rpmF* RNA accumulation in the *ΔyceD* mutant was not affected significantly, the role of *rpmF* in mediating the reduction in 23S rRNA accumulation is unclear. In any case, a specific role for DUF177 in 23S rRNA accumulation is independently supported by evidence that this function is conserved in plastids.

Consistent with the functional association of DUF177 proteins with the large subunit of prokaryotic ribosomes, plastid 23S rRNA levels are sharply decreased in embryo, leaf, and root tissues of the *duf177a* mutant analyzed in the permissive B73 background compared with the wild-type (Fig. 6D). The effect on plastid 23S rRNA accumulation is strongest in tissues that contain chloroplasts and intermediate in root and embryo tissues that contain mixtures of non-photosynthetic plastid types including proplastids, amyloplasts, and leucoplastids, whereas the amyloplast-rich endosperm is least affected (Fig. 6D). In contrast, the plastid 16S rRNA component of the ribosome small subunit and 5S and 4.5S rRNAs of the large subunit showed significant reductions only in embryos and in albino leaves of *duf177a* mutant seedlings where plastid ribosome rRNAs are almost absent (Fig. 6A–C).

Overall, the 23S rRNA content of embryo tissue at 23 DAP is ~25-fold higher on a total RNA basis than in endosperm, suggesting that embryo plastids accumulate substantially more 23S rRNA than amyloplasts (Fig. 6F). Hence, differential requirements of amyloplasts and non-photosynthetic embryo plastids probably contribute to the organ specificity of the *duf177a* phenotype in the non-permissive W22 background (Fig. 2C). The potential for differences in protein translation capacities of non-photosynthetic plastid types has received little attention.

In contrast to the rRNAs, plastid *Rpl32* transcript levels were affected by the *duf177a* mutant only in seed tissues which in maize exclusively contain non-photosynthetic plastids (Fig. 6E). In tobacco, plastid *Rpl32* is transcribed from alternative promoters in photosynthetic and non-photosynthetic plastids, P1 and P2, respectively, conferring differential regulation of plastid *Rpl32* transcription in different plastid types (Vera *et al.*, 1996). Hence, our results are consistent with DUF177A having a role in regulation of RPL32 in non-photosynthetic plastids.

Studies in tobacco indicate that PRPL32 is essential for viability (Fleischmann *et al.*, 2011), whereas L32 is not essential in *E. coli* (Baba *et al.*, 2006) or in *Bacillus subtilis* (Akanuma *et al.*, 2012). The distribution of nuclear *DUF177* genes in plant genomes is not strictly correlated with the presence of plastid-encoded RPL32. In *Populus*, the *Rpl32* gene has been transferred from the plastid genome to the nucleus (Ueda *et al.*, 2007) though *Duf177A* and *Duf177B* genes have been retained. Intriguingly, the absence of *Duf177* from *Chlamydomonas* coincides with the transfer of six large subunit genes (*Prpl2*, *Prpl12*, *Prpl19*, *Prpl22*, *Prpl32*, and *Prpl33*) and three small subunit genes (*Prps11*, *Prps15*, and *Prps16*) to the nucleus within the Chlorophyceae (Maul *et al.*, 2002; Merchant *et al.*, 2007). Although unlike *Chlamydomonas*, *Chlorella* has retained plastid *Prpl32*, loss of plastid *Prpl22* and *Prpl33* genes is common to both *Chlorella* and *Chlamydomonas*. Hence, involvement of DUF177 in regulation of specific *PRPL* genes is not ruled out. While DUF177 proteins have thus far not been directly implicated in regulation of plastid transcription, the ATTED-II (atted.jp) co-expression database indicates that in Arabidopsis *Duf177A* is co-regulated with FLN1, PTAC2, and PTAC15 components of the plastid-encoded RNA polymerase complex (Pfalz and Pfannschmidt, 2013).

### Suppression of emb phenotypes of *duf177a* and plastid translation-related mutants in maize

In W22 maize, *duf177a* blocks embryogenesis at an early transition stage (Fig. 3). While mutant embryos grow slowly through 20 DAP, they retain the ‘ice cream cone’ shape of an early transition stage embryo with radial symmetry about the apical–basal axis. This morphology is characteristic of other maize mutants that disrupt plastid gene expression including *lem1*, *emb8516*, *emb8522*, *tif3*, *why1*, and *emb14* (Ma and Dooner, 2004; Magnard *et al.*, 2004; Sosso *et al.*, 2012; Shen *et al.*, 2013; Zhang *et al.*, 2013; Li *et al.*, 2015). Hence, in a non-permissive genetic background (e.g. W22), plastid gene expression is evidently required for the transition from radial to lateral symmetry associated with formation of the SAM in grass embryo development. The transition stage of embryogenesis is not associated with an obvious change in morphology or differentiation of embryo proplastids (Shen *et al.*, 2013).

We rule out the possibility that genetic redundancy for *duf177a* and other plastid gene expression mutants in the permissive background accounts for suppression of the emb phenotype, because *Duf177A*, *Why1*, *PPR8522*, and *Tif3* are all single-copy genes in the B73 reference genome (Schnable *et al.*, 2009). A more parsimonious explanation is that the B73 background suppresses the requirement for plastid metabolism and/or signaling in embryogenesis rather than by restoring plastid gene expression in the embryo.

In Arabidopsis and other dicots, plastid ribosomes are required for the expression of several plastid-encoded genes (i.e. *accD*, *ycf1*, and *ycf2*) which are essential for plant cell viability (Drescher *et al.*, 2000; Shikanai *et al.*, 2001; Cahoon *et al.*, 2003; Kode *et al.*, 2005). In particular, embryo lethality of Arabidopsis plastid translation mutants has been attributed to the loss of one subunit of heteromeric acetyl-CoA carboxylase activity encoded by the plastid *accD* gene (Bryant *et al.*, 2011). Consistent with this hypothesis, the embryo phenotype of the BSM plastid mTERF RNA processing factor is enhanced by mutations in ACC2, a nuclear-encoded plastid homomeric acetyl-CoA carboxylase (Babiychuk *et al.*, 2011), indicating that acetyl-CoA carboxylase deficiency contributes to embryo lethality of plastid mutants (Parker *et al.*, 2014).

While the *accD* hypothesis *per se* is non-operable in maize and other grasses in which the *accD* gene has been lost from the plastid genome and functionally replaced by nuclear-encoded ACC2 (Konishi and Sasaki, 1994; Konishi *et al.*, 1996; Martin and Herrmann, 1998), similar hypotheses that invoke biochemical complementation of an essential plastid-encoded function by one or more nuclear genes are plausible. In models of this type, suppression of embryo lethality is expected to involve dominant gene action as observed in ACC2 suppression of the *bsm* embryo phenotype in Arabidopsis (Babiychuk *et al.*, 2011; Parker *et al.*, 2014). However, our F_2_ data are consistent with segregation of two recessive suppressor loci in the B73 background that function independently in conditioning albino seedling phenotype (*P*=0.32). A recessive permissive genotype is incompatible with hypotheses that invoke biochemical complementation of an essential plastid-encoded function by nuclear genes. Instead, this pattern implies that embryogenesis is blocked by an active process in the non-permissive background. This interaction is reminiscent of the action of *Inhibitor of Striate 1* (*Isr1*) in maize, which encodes a hydrolase-related protein that inhibits proliferation of albino leaf cells conditioned by the *striate 2* mutant (Joachim and Burnham, 1953; Williams and Kermicle, 1974; Park *et al.*, 2000).

Concievably, lethality due to loss of essential plastid-encoded functions (i.e. *accD*, *ycf1*, and *ycf2*) in Arabidopsis has masked a more fundamental role for plastid signaling in plant embryogenesis. Interestingly, in Arabidopsis, expression of *ACC2* enhances embryo growth, but does not rescue morphogenesis, resulting in larger *bsm* mutant embryos that arrest at a globular stage (Babiychuk *et al.*, 2011; Parker *et al.*, 2014). In contrast, genetic suppression of plastid mutants in B73 maize fully rescues embryo growth as well as morphogenesis to produce albino seedlings (Fig. 4; Sosso *et al.*, 2012; Zhang *et al.*, 2013).

The genetic suppression of emb phenotypes in maize is important for two additional reasons. (i) Our results highlight use of genetic suppression as a tool for classification of emb mutants that facilitates identification of novel genes involved in plastid gene expression and/or signaling. If, for example, the plastid signaling hypothesis is correct, then we anticipate that the class of suppressible emb mutants will include genes involved in the signaling pathway in addition to genes directly implicated in plastid gene expression. (ii) Uncovering the molecular mechanism of suppression is likely to yield new insights into the role of plastids in plant embryogenesis. In addition to B73, other permissive inbred backgrounds include A188, Mo17, and Oh51a (Sosso *et al.*, 2012; Zhang *et al.*, 2013). The effects of genetic background on phenotypes of plastid ribosome mutants were first noted in studies of maize *iojap* (Coe *et al.*, 1988). Depending on the inbred background, *iojap* exhibits a range of phenotypes including emb, albino seedling, and white striped leaves. Hence, the full extent of genetic variation for plastid-dependent embryogenesis in maize has yet to be explored.

### Evolution of ribosome regulation in plastids

Like DUF177, IOJAP proteins are nearly universally conserved, but not essential in bacteria (Häuser *et al.*, 2012). Plant genomes encode two IOJAP proteins (Phytozome, Gramene), one for the chloroplast (Han and Martienssen, 1995), whereas the other is probably targeted to the mitochondria by analogy to IOJAP function in animals (Wanschers *et al.*, 2012). Interestingly, recent studies of human IOJAP homolog C7orf30 show that mitochondrial ribosomal protein L32 levels are reduced in C7orf30 knockdown lines, implying an association between the two proteins (Fung *et al.*, 2012). While the functional relationship between DUF177A and IOJAP in plastid ribosome formation is not yet known, it is striking that in both cases key mechanisms controlling plastid ribosome assembly evidently evolved from non-essential and still enigmatic pathways that regulate ribosome functions in bacteria. An intriguing hypothesis emerging from studies of IOJAP in mammalian mitochondria is that IOJAP plays a role in differentiating specialized subsets of ribosomes involved in assembly of membrane complexes (Fung *et al.*, 2012). Conceivably, DUF177A may have an analogous function in plastids where translational regulation plays a central role in differentiation (Sun and Zerges, 2015).

## Supplementary data

Supplementary data are available at *JXB* online.

Figure S1. Unrooted tree of DUF177 protein sequences.

Figure S2. Amino acid alignment of plant DUF177 domain sequences.

Figure S3. Dry weight of wild-type and *duf177a* mutant endosperm at maturity.

Data S1. Alignment of plant DUF177 proteins by MUSCLE.

Data S2. Alignment of amino acid sequences of the DUF177 domain by MUSCLE

Supplementary Data

## Abbreviations:

Col-0: Columbia-0
DAP: days after pollination
DUF: domain of unknown function
emb: embryo lethal
PRP: plastid ribosomal protein
SAM: shoot apical meristem.
