# Supplementary material for: Essential role of conserved DUF177A protein in plastid 23S rRNA accumulation and plant embryogenesis
Source: J Exp Bot. 2016 Aug 29;67(18):5447–60. doi: 10.1093/jxb/erw311 (PMC5049393; doi:10.1093/jxb/erw311)
Supplement: Supplementary Data [file supp_67_18_5447__index.html]

Essential role of conserved DUF177A protein in plastid 23S rRNA accumulation and plant embryogenesis — Supplementary Data 

# Essential role of conserved DUF177A protein in plastid 23S rRNA accumulation and plant embryogenesis

## Supplementary Data

Data files

- Supplementary\_Figures\_S1\_3.pdf - Supplementary Data
- Supplementary\_Data\_S1.pdf - Supplementary Data
- Supplementary\_Data\_S2.pdf - Supplementary Data
