# Supplementary material for: Essential role of conserved DUF177A protein in plastid 23S rRNA accumulation and plant embryogenesis
Source: J Exp Bot. 2016 Aug 29;67(18):5447–60. doi: 10.1093/jxb/erw311 (PMC5049393; doi:10.1093/jxb/erw311)
Supplement: Supplementary Data [file supp_erw311_Supplementary_Figures_S1_3.pdf]

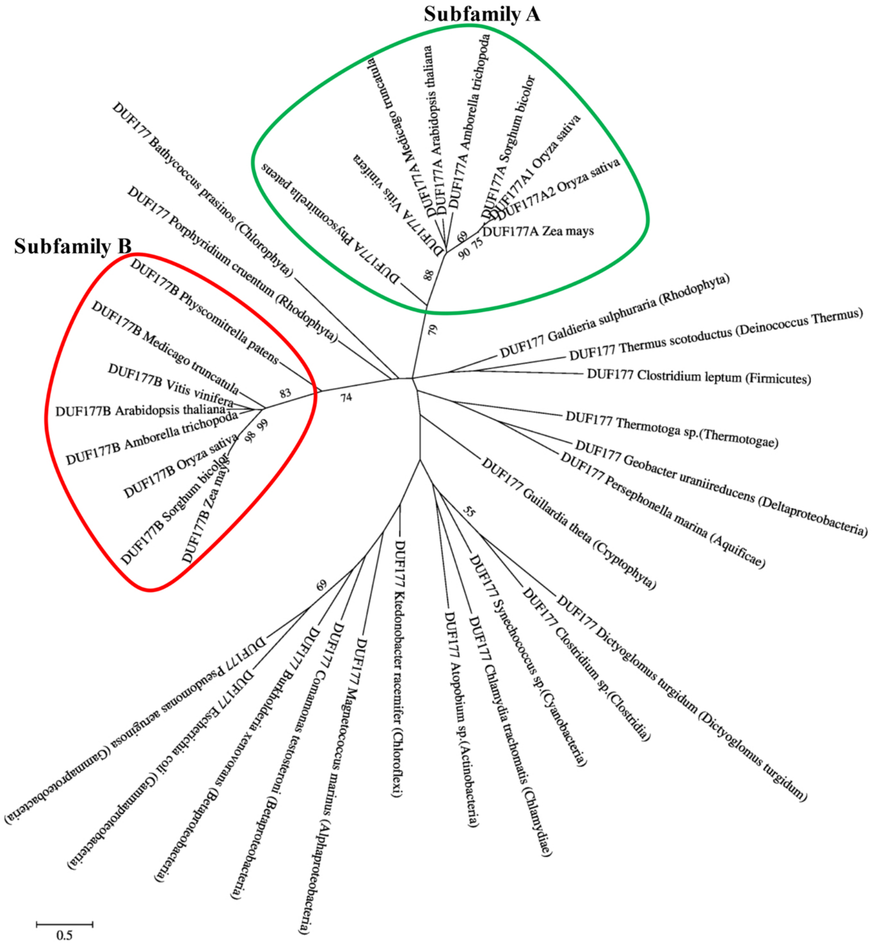

Supplementary Fig. S1. Unrooted tree of DUF177 protein sequences.

Amino acid sequences of DUF177 domain from representative species of plant, algae, and bacteria (Data S2) were aligned by MUSCLE and used to construct a maximum likelihood tree based on the JTT matrix-based model by MEGA6 (Tamura et al., 2013). Bootstrap support was based on 1000 iterations. Bootstrap support values are shown for nodes with 50% or greater. Plant species include *Physcomitrella patens, Amborella trichopoda, Medicago truncatula*, *Arabidopsis thaliana*, *Vitis vinifera*, *Oryza sativa*, *Sorghum bicolor*, and *Zea mays.* Bacterial DUF177 domains from Proteobacteria, Actinobacteria, Deinococcus Thermus, Thermotogae, Aquificae, Dictyoglomia, Firmicutes, Clostridia, Cyanobacteria, and Choroflexi, and DUF177 domains from *Guillardia theta,* red algae (*Galdieria sulphuraria* and *Porphyridium cruentum*) and green algae (*Bathycoccus prasinos*) are included in the tree.

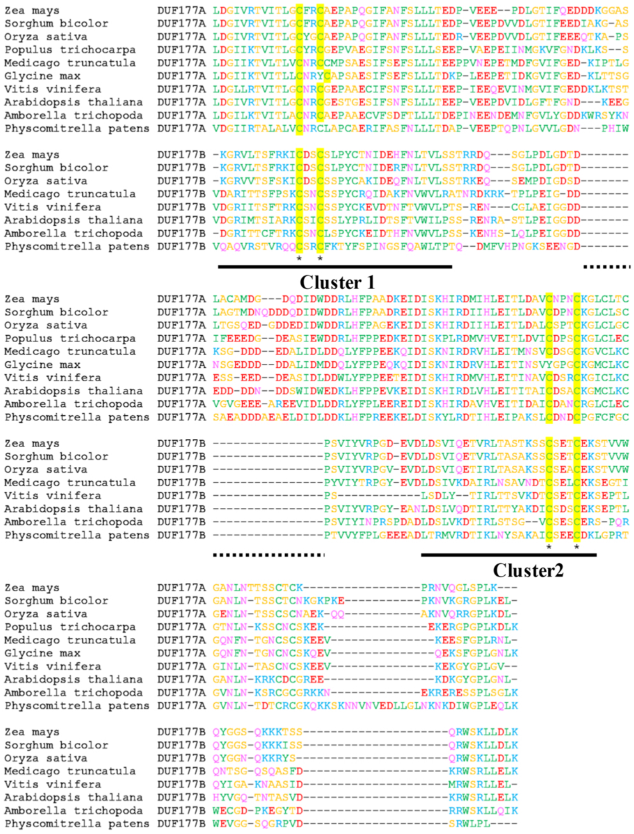

Supplementary Fig. S2. Amino acid alignment of plant DUF177 domain sequences.

DUF177 domain sequences from proteins of *Physcomitrella patens, Amborella trichopoda, Medicago truncatula*, *Arabidopsis thaliana*, *Vitis vinifera*, *Populus trichocarpa*, *Glycine max*, *Oryza sativa*, *Sorghum bicolor*, and *Zea mays,* were aligned by MEGA6 (Tamura et al., 2013). The two clusters of conserved amino-acid residues are indicated by solid lines and the highly conserved cysteine residuals in the two cysteine motifs are color highlighted in yellow and indicated by asterisks. The region underlined by broken line (amino acids 53-76) represents the plant DUF177A-specific insertion missing from plant DUF177B domains. Small nonpolar, orange; hydrophobic, green; polar, magenta; negatively charged, red; and positively charged, blue.

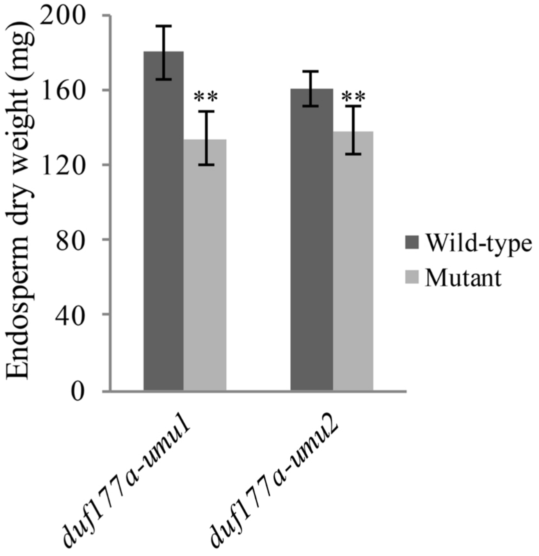

Supplementary Fig. S3. Dry weight of wild-type and *duf177a* mutant endosperm at maturity.

**, *P*-value<0.01. Error bars show standard deviation. n=30
