# Supplementary material for: Essential role of conserved DUF177A protein in plastid 23S rRNA accumulation and plant embryogenesis
Source: J Exp Bot. 2016 Aug 29;67(18):5447–60. doi: 10.1093/jxb/erw311 (PMC5049393; doi:10.1093/jxb/erw311)
Supplement: Supplementary Data [file supp_erw311_Supplementary_Data_S1.pdf]

```
>DUF177A1_Oryza_sativa
----------------------------------------------------MIGFGIGGVAGALPLVLRRTRTCTPPPP
----------------------------------------------------------------------MAMTPLH--H
TCSFFFRPQPSP----------------SLSHSPCAAAADDDFF----TVDYD-----PEEEK-----------------
-----------------------EEDEEGSPWEGAVVYRRDASVHHLEYATTLERLGLGDLSSTHSRARAATMGILILSS
TNLTGTKDDDTPVLVSLDVARR-RGRLRLDGIVRTVITLGCYGCAEPAPQGIFANFSLLLTEDRV-EEP-DVVDLGTIFE
EEQTKAPSLTGSQED-GDDEDIDWDDRLHFPAGEKEIDISKHIRDIIHLEITLDALCSPTCKGLCLGCGENLN-TSSCSC
NAEKQQ--AKNVQR----------RGPLKDLLKPLQR--------

>DUF177A_Sorghum_bicolor
-------------------------------------------------------------MGRARSCT-----------
--------------------------------------------------------------------------------
--------------------------------------------------------------------------------
--------------------------------------RRDAAVHHLEYATTLERLGLGDLSSPDSRARAANLGLGLSGG
GQDGTAAQSQTPVLVSLDVTRR-RGRLRLDGIVRTVITLGCFRCAEPAPQGIFANFSLLLTEDPV-EEP-DVVDLGTIFE
EDIAKGASLAGTMDNQDDDQDIDWDDRLHFPAADREIDISKHIRDIIHLEITLDAVCDPNCKGLCLSCGANLN-TSSCTC
NKGKPK-EPKNVKG----------RGPLKELLKPMQKR-------

>DUF177A2_Oryza_sativa
--------------------------------------------------------------------------------
----------------------------------------------------------------------LVGETRRRRE
RPALLLPVTVPP--------------------------------------------------------------------
--------------------------------------------------STVDRPPLRRLAAS----------------
-------------TLPPTASRATSPRRRRPELQPPPPRVNC--CAEPAPQGIFANFSLLLTEGRV-EEP-DVVDLGTIFE
EEQTKAPVLTGSQED-GDDEDIDWDDRLHFPAGEKEVDISKHIRDIIHLEITLDALCSPTCKGLCVGCGENLN-TSSCSC
NTEKQQAKAKNVQR----------RGPLKRPVETTAKVM------

>DUF177A_Physcomitrella_patens
MIPHIQLEISENNPFYIHRLVIQLEPSGIRSTQDRRSSSSKRPVVAGDEARDLVGGAWTTSSAMATASSTSLPAAAPQQP
NVDSALGLQVHQLEVACAELQSLHETRAVYEKRGGVFFRTTVKAATKSQQSLNQLVHVMPAGFAEVGALDPDISFHH--R
PRLHHLCAESSQHGRCEVASRASSYKQGGWVRVSNVACLGVLGTSCSSRRQYLRCVNSRQDTTSNERFRDNSNFSPALRP
FISRGHTRVLRQHNALPANPGVVEEEEQGTPWEGAIIFKRSAAQAQFEYVTTLERLGLTRFSSDKAMTLAFDMGL-----
GKVEVDGIVGTPVLISLDCSKEGRNDIRVDGIIRTALALVCNRCLAPCAERIFASFNLLLTDAPV-EEP-TQPNLGVVLG
DNPHI--WSAEADDDAEAELDIDLDDKLHFPREEKELDISKYLRDTIHLEIPAKSLCDNDCPGFCFGCGVNLN-TDTCRC
GKQKKSKNNVNVEDLLGLNKNKDIWGPLEQLKKQLEEQDRSDDSL

>DUF177A_Amborella_trichopoda
------------------------------------------------------------MAFSTLSCPLSPHLHNPQFS
SKPSAKT---------------------------------------------------------------PSFNQLESHL
KPSKTLISSYSS---------ISKPYTTRPIISPSKGLISISSLHCTSSETVS-----HESTE-----------------
-----------------------EVEDEGSPWEGAVVYKRDSSVTHLEYCTTLERLGLGKLSSDVSNSRASSMGI---RV
TKGVTEYSSGTPVQISIDVYRR-KQDIKLDGILKTVITLACNRCAEPAAECVFSDFTLLLTDEPINEEN-DEMNFGVLYG
DDKWRSYKNVGVGEEEAREEVIDLDDRLYFPLEEREIDISKHIRDAVHVEITIDAICDANCRGLCLECGVNLN-KSRCGC
GRKKNE---KRERE----------SSPLSGLKHQTYKQ-------

>DUF177A_Arabidopsis_thaliana
----------------------------------------------MSLVCSLSCVAPLPQTKQSRPSFLKLETCTLSLS
SPAGY-----------------------------------------------------------------PNFTTGI--R
KHISYLFTEPIK----------------LPRLAKSRILVSQESFTETSTIDMD-----WEDQE-----------------
-----------------------EIEDTGSPWEGSVMYRRNASVTHVEYCTTLERLGLGRLSTDVSKKRASAMGL---RV
TKDVKDYPDGTPVQVSVDVIRK-KKKLRLDGIVRTVITLGCNRCGESTGESIFSNFSLLLTEEPV-EEP-DVIDLGFTFG
NDKEE-----GEDDDDNDDSWIDWEDKLHFPPEVKEIDISKHIRDLVHLEITITAICDSACKGMCLKCGANLN-KRKCDC
GREEKD------KG----------YGPLGNLREQMQQKEGLRN—

>DUF177A_Zea_mays
--------------------------------------------------------------MYYPQPTVSLAAAVALLR
----------------------------------------------------------------------PSLRRHS--Q
RASSLLRSSTPP----------------PWVSARRRTAVSDDFF----TVELDATGVEPEPDS-----------------
-----------------------IDDGLPSPWEGAVVYRRDAAVQHLEYASTLERLGLGDLSSPDSRARAADLGL--AGG
TLDSTGAQPRTPVLVSVDVTRR-RGRLRLDGIVRTVITLGCFRCAEPAPQGIFANFSLLLTEDPVEEEP----DLGTIFQ
EDDDKGGASLACAMD--GDQDIDWDDRLHFPAADKEIDISKHIRDMIHLEITLDAVCNPNCKGLCLTCGANLNTTSSCTC
KP-------RNVQG----------LSPLKGVFK------------

>DUF177B_Physcomitrella_patens
------------------------MFPSSQGQRWTHSLYSTHFVSVDNAGAGVVKLRVLQYSQAVDMANVLLHTPRPSFS
TPWGVSDSRWC-----------------------------------------------------------LGFESRCGAG
RVMGWCCRSGRV----------------VVCTTGQHPPEPRKEFSHVESKPVSKKLPKTNKKK-----------------
------------EGRERVLPVLITVNRSDGKW--ADSWNTE-------QVTTLKDLNLEDISTDS---------------
SFQGPGKVPKDLVHVELAVQKS-GWGFFVQAQVRSTVRQQCSRCFKTYFSPINGSFQAWLT--PTQDMF-VHPNGKSEEN
GDPTV---------------------VYFPLGEEEADLTRMVRDTIKLNYSAKAICSEECDKLG---------PRTWEV
GGSQGR---PVDSR----------WLPLLKAKHNL----------
```

```
>DUF177B_Oryza_sativa
---------------------------------------------------------------MARACSPALRLQLPPNP
PITPQL----------------------------------------------------------------PSCRTHSAGA
RCRGFAAAHSQP------------PAAGRPDEPAAEPSPKQPEIAQTQNLRRSRRRG-PGSRQ-----------------
-----------------------SLVSVGTSCGGGDQWSSD-------FDLTLRQLHLDDLIED----------------
-----GQNDDADVLVHLLVQQHTQFGMSIKGRVVTSFSKICDSCSSPYCAKIDEQFNLTVLSSTRKEQS-EMPDIG---D
SDPSV----------------------IYVRPGV-EVDLDSVIQETIRLTASAKSSCSEACEKS----------TVVWQY
GGNQKK---RYSQR----------WSKLLDLKKTLDKAAN-----

>DUF177B_Amborella_trichopoda
--------------------------------------------------------------------------------
--------------------------------------------------------------------------------
--------------------------------------------------------------------------------
--------------------------------------------------------------------------------
-------------MVSLSIQKHVGFGYSIDGRITTCFTRKCSNCLSPYCKEIDTHFNVWVLPSSKENHSLQLPEIG---G
DDPSV----------------------IYINPRSPDADLDSLVKDTIRLSTS--GVCSESCERS----------PQRWEC
GDPKEG---YTDRR----------WSKLLQIKVTA----------

>DUF177B_Sorghum_bicolor
---------------------------------------------------------------MARACNRALRLLPNSIA
----------------------------------------------------------------------TQLASSRSGA
RFRSLAVHAQLS------------------TEDDAYSTEPLKKVQVTQSLRRTRRRGTGGARQ-----------------
-----------------------SLVSVGTSCGGGDQWSSD-------IELTLRQLHLDDLIED----------------
------GQRDADVLVHLLVQQHTQFGMSIKGRVLTSFRKICDSCSLPYCTNIDERFNLTVLSSTRRDQS-GLPDLG---D
TDPSV----------------------IYVRPGD-EVDLDSVIQETVRLTASAKSSCSETCEKS----------TVVWQY
GGSQKK--KISSQR----------WSKLLDLKKTLDKAPK-----

>DUF177B_Zea_mays
---------------------------------------------------------------MARACNHALRLLPNSIA
----------------------------------------------------------------------TQLACSGGST
RCRNLSVHAQLS-----------------TQDDAYTTEPPLKKAQVTQSLRRTRRRAPGGARQ-----------------
-----------------------SLISVGTSCGGGDQWSSD-------IALTLRQLRLDDLVED----------------
------GQRDADVLVHLLVQQHTQFGMSIKGRVLTSFRKICDSCSLPYCTNIDEHFNLTVLSSTRRDQS-GLPDLG---D
TDPSV----------------------IYVRPGD-EVDLDSVIQETVRLTASTKSSCSETCEKS----------TVVWQY
GGSQKK--KTSSQR----------WSKLLDLKKTLDKAPK-----

>DUF177B_Arabidopsis_thaliana
---------------------------------------------------------------MDVRCLISPNLLNSKIK
----------------------------------------------------------------------VSGNTHH--L
PFSSLSKKHQAS----------------SPIQAAINGGGSSKTVKRLI--------------------------------
-----------------------TLSPSEGKWNGN--WKTQ-------YDVSLRDLQLQDLVED----------------
------GPPNSRVSVDLSVQRHASMGLSVDGRIMTSIARKCSICSSLYPRLIDTSFTVWILPSSRENRASTLPEIG---G
DDPSV----------------------IYVRPGY-EANLDSLVQDTIRLTTYAKDICSDSCEKSE---------PTLHYV
GQTNTA---SVDKR----------WSRLLELKKK-----------
```
