# Supplementary material for: Essential role of conserved DUF177A protein in plastid 23S rRNA accumulation and plant embryogenesis
Source: J Exp Bot. 2016 Aug 29;67(18):5447–60. doi: 10.1093/jxb/erw311 (PMC5049393; doi:10.1093/jxb/erw311)
Supplement: Supplementary Data [file supp_erw311_Supplementary_Data_S2.pdf]

>DUF177 Chlamydia_trachomatis (Chlamydiae)
-QGTITARVHAECSRCLMPLHRDWSVDVCAFFAQV---ESKGGARSNNSKFNRSNGDNDDL------------ED----A
DIWDEGDDSGNVYPLVGGGDF-ADIEALIRDTMVSELPLKPLCEPDCKGLCSQCGENLN-EHP-NHHHDIT---------
--------DIRFAALEGLK
>DUF177 Porphyridium cruentum (Rhodophyta)
LRGRVSTVLNCACDRCLDEFPFQSRGEFELWLATR---EDLV---------PMIDGPDDTR------------AD-----
---------EAVEKFTDDILA-VDLTPHVYDAIMLSVPIKKLCREDCPGL------------------------------
----
>DUF177 Galdieria sulphuraria (Rhodophyta)
-KSRVNTCLTCECDRCLNPFRLAVEGYFQLLLSSN---PNVQ-----EVDHSVVGGEEKET------------QQLTSTT
VVGFEDLEMEVWEPFTSDVDQ-VSLYSHVYDSVMLSLPSKILCDESCPGIP-----------------------------
----
>DUF177 Thermus scotoductus (Deinococcus Thermus)
LSGEVEGVVLMECRRCLKPTPTPIHAHFQHLLRYQ---QGLE---------EVVFHEEKEE-------------------
------------EYYAFGEPD-LDLLPFLTEAFVTEMPYTVLCEEGCKGLCPVCGADRN-LVD-CGHEPEV---------
--------HHPLLGLKDL-
>DUF177 Atopobium sp.(Actinobacteria)
-TGMLNAHVVGSCDRCLEKAEFDVAGEVDEYFLFH----EPD---------PASLGDDEDE------------ID-----
------------FALVSADQT-IDLSEALSSALLMETPFVVLCKPDCRGLCPVCGANLN-EED-CGHAAQIEQDRLS---
--------SSPFAKLKELK
>DUF177 Dictyoglomus turgidum (Dictyoglomus_turgidum)
-KGSLKTKIKLVCSRCLREFPYELEAKIQEIYLWD---IPIQ--------RNISPGEIIEL------------KD-----
----------EDFKFVLEKES-LFLDPLVEDIIRLNIPVKPLCRPDCKGLCPGCGQDLN-LGE-CECSKKIQV-------
--------DPRWEPLMKF-
>DUF177 Synechococcus_sp.(Cyanobacteria)
-SAEARTIVTLTCHRCLQQFNHRLHLEVEEIILIR----DPE-----PLPLELELQDEEDL-------------------
------------LESLPPNGE-LDVEDWMYQHLHLEMPRQLPCRPDCPGIAVEATAPTA---------------------
--------DPRWAALASL-
>DUF177 Thermotoga sp.(Thermotogae)
--GYVHTAVEHPCARCLEPARVEIRGVIEALYLPE---SMRK---------NVKEEKLESL------------KN-----
------------IIYYHETE--FDLSERIIEAIVVAIPEKVLCKPDCKGLCPYCGANLN-EEPDHKCDKIPVV-------
--------DSRFEILAELK
>DUF177 Clostridium sp.(Clostridia)
VQGTIVANVKLVCDRCLKDFVKKMKVDVDETYLLG---HLIA-------NEDAHSGQELEL------------KD-----
---------GDFVTELTGTDE-VDIDDLIYQSVTLNIPNPCVCDINCNG-----------DAEMEKYMKKEIS-------
--------DPRLEVFKNLK
>DUF177 Geobacter uraniireducens (Deltaproteobacteria)
-AGKVETAVMMNCSLCLGEYDTEIASSFTVFYTKA---SGMA------LDEEVELAEEDLI-------------------
-------------SKSYEGDE-IDFAPEVAEQVIMEIPFKPLCKEDCAGLCSKCGVNLN-ETA-CNCDRSEG--------
--------GFKFGALKGIK
>DUF177 Ktedonobacter racemifer (Chloroflexi)
VDGWVDLTVELECTRCLKHFEQPQHVDFEERFYPT---VDVV------TGIPVEPVDAEDA-------------------
--------------FPIDDHHLVDLTEAIRQQVLLAIPIVTLCREDCAGLCAQCGKDLN-EGP-CECQPEP---------
--------DARFSVLKTL-
>DUF177 Clostridium leptum (Firmicutes)
-KADVAFDFRCPCDRCAAETQRAYRFSFQHVLVNA------------------LNDEENDT-------------------
-------------FLLVENES-IELDDLLREDILLELPTKFLCKPDCKGLCPQCGKNLN-EGK-CGCSARQV--------
--------DPRLEILKKL-
>DUF177 Persephonella marina (Aquificae)
-SLTINSDIKLECGRCLESFVMDLKGTSSIFLSKK------------KLDGDSELHEEDLI-------------------
------------VEYLEDEEH-FNVSELLREEILVKTPMKPLCDEDCKGICPVCGSNRN-ENP-CNCEQEMVRE------
--------ESPFAKLKTL-
>DUF177 Guillardia theta (Cryptophyta)
LKVHMQSHLLCTCNRCLEEFHLPISSDFSLVLASR---RKLCQIAKRRADDVSEISENKDL------------VD-----
---------DSIIDFSAGINY-IDLDPEVGESLGGAIPLRKVCSPTCKGRCATCGQNLNLPGNTCRCRRVPQ--------
--------EER--------
>DUF177 Bathycoccus prasinos (Chlorophyta)
LQAEITGHIRCHCDACDGKFNLPVKSGFKLFLDEH------------ATAFGDVSGDLEIV-------------------
-------------PFPRSTEH-VDLTSVARSWIEMNLKEEFLCDE-------------------CGEND-----------
----

```
>DUF177A2 Oryza sativa
---------------CAEPAPQGIFANFSLLLTEGRV-EEPD-----VVDLGTIFEEEQTK-APVLTGSQED-GD-----
---DEDIDWDDRLHFPAGEKE-VDISKHIRDIIHLEITLDALCSPTCKGLCVGCGENLN-TSS-CSCNTEKQQA------
-------------------
>DUF177A1 Oryza sativa
LDGIVRTVITLGCYGCAEPAPQGIFANFSLLLTEDRV-EEPD-----VVDLGTIFEEEQTK-APSLTGSQED-GD-----
---DEDIDWDDRLHFPAGEKE-IDISKHIRDIIHLEITLDALCSPTCKGLCLGCGENLN-TSS-CSCNAEKQ-QAKN---
--------VQRRGPLKDL-
>DUF177A Medicago truncatula
LDGIIKTVLTLVCNRCCMPSAESIFSEFSLLLTEEPPVNEPE-----TMDFGVIFGEDKIP---TLGKSGDDDED-----
----ALIDLDDQLYFPPEEKQ-IDISKNIRDRVHLEITMNSVCDSGCKGVCLKCGQNFN-TGN-CSCSKEEVK-------
--------EESFGPLRNL-
>DUF177A Sorghum bicolor
LDGIVRTVITLGCFRCAEPAPQGIFANFSLLLTEDPV-EEPD-----VVDLGTIFEEDIAK-GASLAGTMDNQDD-----
---DQDIDWDDRLHFPAADRE-IDISKHIRDIIHLEITLDAVCDPNCKGLCLSCGANLN-TSS-CTCNKGKPKEPKN---
--------VKGRGPLKEL-
>DUF177A Arabidopsis thaliana
LDGIVRTVITLGCNRCGESTGESIFSNFSLLLTEEPV-EEPD-----VIDLGFTFGNDKEE------GEDDDDND-----
---DSWIDWEDKLHFPPEVKE-IDISKHIRDLVHLEITITAICDSACKGMCLKCGANLN--KRKCDCGREEK--------
--------DKGYGPLGNL-
>DUF177A Physcomitrella patens
VDGIIRTALALVCNRCLAPCAERIFASFNLLLTDAPV-EEPT-----QPNLGVVLGDNPHI---WSAEADDDAEA-----
---ELDIDLDDKLHFPREEKE-LDISKYLRDTIHLEIPAKSLCDNDCPGFCFGCGVNLN-TDTCRCGKQKKSKNNVNVE-
DLLGLNKNKDIWGPLEQLK
>DUF177A Amborella trichopoda
LDGILKTVITLACNRCAEPAAECVFSDFTLLLTDEPINEEND-----EMNFGVLYGDDKWRSYKNVGVGEEEARE-----
----EVIDLDDRLYFPLEERE-IDISKHIRDAVHVEITIDAICDANCRGLCLECGVNLN--KSRCGCGRKKNEKR-----
--------ERESSPLSGLK
>DUF177A Vitis vinifera
LDGLLRTVITLGCNRCGEPAAECIFSNFSLLLTEEPI-EEQE-----VINMGVIFGEDDKL--KTSTESSEE-DD-----
---EASIDLDDWLYFPPEETE-IDISKHIRDMVHLEITINAVCDSRCKGICLKCGINLN-TAS-CNCSKEEVK-------
--------EKGYGPLGV--
>DUF177A Zea mays
LDGIVRTVITLGCFRCAEPAPQGIFANFSLLLTEDPVEEEPD--------LGTIFQEDDDKGGASLACAMDGDQD-----
------IDWDDRLHFPAADKE-IDISKHIRDMIHLEITLDAVCNPNCKGLCLTCGANLN-TTSSCTCK------------
--------PRNVQGLSPLK
>DUF177B Oryza sativa
-KGRVVTSFSKICDSCSSPYCAKIDEQFNLTVLSS---TRKE-----QSEMPDIGDSDPSV-------------------
-------------IYVRPGVE-VDLDSVIQETIRLTASAKSSCSEACEKSTV--------VWQYGGNQKKRY--------
--------SQRWSKLLDLK
>DUF177B Arabidopsis thaliana
VDGRIMTSIARKCSICSSLYPRLIDTSFTVWILPS---SREN----RASTLPEIGGDDPSV-------------------
-------------IYVRPGYE-ANLDSLVQDTIRLTTYAKDICSDSCEKSEP--------TLHYVGQTNTASV-------
--------DKRWSRLLELK
>DUF177B Zea mays
-KGRVLTSFRKICDSCSLPYCTNIDEHFNLTVLSS---TRRD-----QSGLPDLGDTDPSV-------------------
-------------IYVRPGDE-VDLDSVIQETVRLTASTKSSCSETCEKSTV--------VWQYGGSQKKKTS-------
--------SQRWSKLLDLK
>DUF177B Vitis vinifera
VDGRIITSFTRKCSNCSSPYCKEVDTNFTVWVLPT---SREN------CGLAEIGGDDPSL-------------------
----------------------SDLY-----TIRLTTSVKDTCSETCEKSEP--------TLQYIGAKNAASI-------
--------DMRWSRLLEL-
>DUF177B Sorghum bicolor
-KGRVLTSFRKICDSCSLPYCTNIDERFNLTVLSS---TRRD-----QSGLPDLGDTDPSV-------------------
-------------IYVRPGDE-VDLDSVIQETVRLTASAKSSCSETCEKSTV--------VWQYGGSQKKKIS-------
--------SQRWSKLLDLK
>DUF177B Medicago truncatula
VDARITTSFPSKCSNCSSPYCRQIDAKFNVWVLRA---TNRD---KRKTPLPEI-GDDPYV-------------------
-------------IYTRPGYE-VDLDSIVKDAIRLNSAVNDTCSELCKKSEGT-------IQNTSGQSQASF--------
--------DKRWSRLLELK
```

```
>DUF177B Amborella trichopoda
-DGRITTCFTRKCSNCLSPYCKEIDTHFNVWVLPS---SKEN----HSLQLPEIGGDDPSV-------------------
------------IYINPRSPD-ADLDSLVKDTIRLSTSGV--CSESCERSPQ--------RWE-CGDPKEGYT-------
--------DRRWSKLLQIK
>DUF177B Physcomitrella patens
VQAQVRSTVRQQCSRCFKTYFSPINGSFQAWLTPT---QDMF----VHPNGKSEENGDPTV-------------------
------------VYFPLGEEE-ADLTRMVRDTIKLNYSAKAICSEECDKLGPR-------TWEVGGSQGRPV--------
--------DSRWLPL----
>DUF177 Escherichia coli (Gammaproteobacteria)
LNGDAKVTVTLECQRCGKPFTHQVYTTYCFSPVRS---DEQA---------EALPEAYEPI-------------------
--------------EVNEFGE-IDLLAMVEDEIILALPVVPVHDSE---HCEVSEADMV-FGELPEEAQK----------
--------PNPFAVLASLK
>DUF177 Pseudomonas aeruginosa (Gammaproteobacteria)
-HMQLDTEVSMVCQRCLDAAAIPVHGEYTYAILRE---GQSA---------DGLPKGYDAL-------------------
---------------EVGEEP-LDLLALVEDELLLALPIVPAHDPE---VCQ-HPAGFVVEDEPESSEVEDKR-------
--------PNPFSVLAQLK
>DUF177 Comamonas testosteroni (Betaproteobacteria)
-HLSAEVALPLTCQRCLTEADIPLYVDRSFRFVPD---EATA----------ELEDDDSDE-------------------
-------------DVLALSSE-FNLLELIEDELLMEVPVVPRH-EVCP-VPVKLEVSDDAFEQA-NEQK-----------
--------ENPFAVLQSL-
>DUF177 Burkholderia xenovorans (Betaproteobacteria)
-RLAIHGAAWLECQRCMTPYLQAFNVDATYRVVNT---EAEA---------EEFPLDEDEV-------------------
-------------EVIVGSNH-FDLIDLIEEELLLSLPLVPKHE-VCPEVHESLVSGVAGAEGEEDESDESAPDEAGKGD
EPER----PSPFAALQSLK
>DUF177 Magnetococcus marinus (Alphaproteobacteria)
VTGTVTATVSMNCSRCLVDFERLLEGDVERWYATG---VDPN-----NGSMGELAVTDETV-------------------
---------------YLEDDL-FTLAPLADEELLLHLPMVPLCGEGCKGICAQCGANLN-EGP-CGCDDGPQ--------
--------DSPFAALKLLK
```
